# A Microfluidic Cell Co-Culture Chip for the Monitoring of Interactions between Macrophages and Fibroblasts

**DOI:** 10.3390/bios13010070

**Published:** 2022-12-31

**Authors:** Pengcheng Li, Feiyun Cui, Heying Chen, Yao Yang, Gang Li, Hongju Mao, Xiaoyan Lyu

**Affiliations:** 1 Department of Orthopedics, West China Hospital, West China School of Nursing, Sichuan University, Chengdu 610041, China; 2 School of Basic Medical Sciences, Harbin Medical University, Harbin 150081, China; 3 The Ministry of Education Key Laboratory of Clinical Diagnostics, School of Laboratory Medicine, Chongqing Medical University, Chongqing 400016, China; 4 Department of Dermatology, West China Hospital, Sichuan University, Chengdu 610041, China; 5 Laboratory of Dermatology, Clinical Institute of Inflammation and Immunology, Frontiers Science Center for Disease-Related Molecular Network, West China Hospital, Sichuan University, Chengdu 610041, China; 6 Key Laboratory of Optoelectronic Technology and Systems, Ministry of Education, Defense Key Disciplines Lab of Novel Micro-Nano Devices and System Technology, Chongqing University, Chongqing 400044, China; 7 State Key Laboratory of Transducer Technology, Shanghai Institute of Microsystem and Information Technology, Chinese Academy of Sciences, Shanghai 200050, China

**Keywords:** microfluidic chips, organ-on-a-chip, wound healing, cell co-culture, macrophages

## Abstract

Macrophages and fibroblasts are two types of important cells in wound healing. The development of novel platforms for studying the interrelationship between these two cells is crucial for the exploration of wound-healing mechanisms and drug development. In this study, a microfluidic chip composed of two layers was designed for the co-culturing of these two cells. An air valve was employed to isolate fibroblasts to simulate the wound-healing microenvironment. The confluence rate of fibroblasts in the co-culture system with different macrophages was explored to reflect the role of different macrophages in wound healing. It was demonstrated that M2-type macrophages could promote the activation and migration of fibroblasts and it can be inferred that they could promote the wound-healing process. The proposed microfluidic co-culture system was designed for non-contact cell–cell interactions, which has potential significance for the study of cell–cell interactions in biological processes such as wound healing, tumor microenvironment, and embryonic development.

## 1. Introduction

Skin wound-healing is a complex and highly coordinated process that can be divided into three stages: (1) hemostasis and inflammatory stage, (2) proliferative stage, and (3) matrix deposition and remodeling [1,2,3]. Fibroblasts play a critical role in these processes as they promote wound contraction, wound closure, and produce extracellular matrix (ECM) components [4]. Although over-activation of fibroblasts can lead to fibrosis and scarring [5], they have many vital effects, such as the reconstruction of connective tissue including the dermis of the skin [6,7], muscle reparation, and as a source of adipocyte progenitor cells [8]. Therefore, the degree of fibroblast activation may determine the ultimate healing fate. As another key cell type in wound healing, macrophages contribute to all stages of tissue repair. They provide essential inflammatory and debris clearance functions during early trauma before giving way to functions that support healing during the regeneration phase. During healing, M2 macrophages secrete essential factors such as platelet-derived growth factor α (PDGF-α), interleukin-10 (IL-10), transforming growth factor β1 (TGF-β), fibronectin (FN), resistin-like molecule α (RELM-α), and vascular endothelial growth factor (VEGF) that activate fibroblasts and drive tissue regeneration [9,10,11,12,13,14]. At the same time, fibroblasts are able to induce the transformation of M1 macrophages into M2 type to prevent excessive inflammation and promote wound healing. Therefore, elucidating the interrelationship between these two cells is crucial for the exploration of wound-healing mechanisms and drug development.

At present, scratch assay and transwell migration assay are commonly used in in vitro experimental methods to observe different stimulus factors on cellular regulation, as well as study interactions between cells [15]. The advantages of the two methods are simple operation and suitable for routine laboratory operations. However, the scratch assay is not conducive to the observation of the interaction between different cells, and the transwell migration assay is not suitable for the dynamic observation of cell migration. Both of them are low throughput and the types of cells that can be observed at the same time are limited [16,17]. Therefore, these methods are not competent for observing the regulatory effect of macrophages with different polarities on a variety of skin intrinsic cells. For in vivo experimental methods, they are relatively complex, time-consuming, and expensive. It is also impossible to independently observe the effects of macrophage-secreted factors on different cells and find the main effector cells regulated by macrophage-secreted factors.

Microfluidics is a set of artificially fabricated miniature systems that operate on small flow volumes [18]. This system can ensure miniaturization, integration, automation, and parallelism in analytical processes [19]. The most prominent advantage of microfluidics for cell culture is that they can better simulate the physiological conditions of in vitro culture [20]. Moreover, the design of microfluidic devices is flexible and the experimental operation is highly controllable [21]. Meanwhile, because the dosage of related reagents is small, the demand for cells is low and it is conducive to the observation of single cells [22]. Using a microfluidic chip, the entire analysis process can be detected dynamically in real-time, and the observation results are conducive to effective statistical analysis [23]. At present, microfluidic technology is involved in many aspects of cell culture, such as cell co-culture, physiological microenvironment construction, neural cell development, single-cell metabolism, and cell secretion [24]. The use of microfluidic chambers for cell co-culture has been widely used to observe the interaction between cells, such as in the study of neuromuscular junctions, tumor neural invasion, and the interaction between tumor cells and stromal cells [25,26,27]. However, the application of microfluidic chips in the research on cellular and molecular mechanisms related to skin damage repair is rarely reported.

**Scheme 1:**
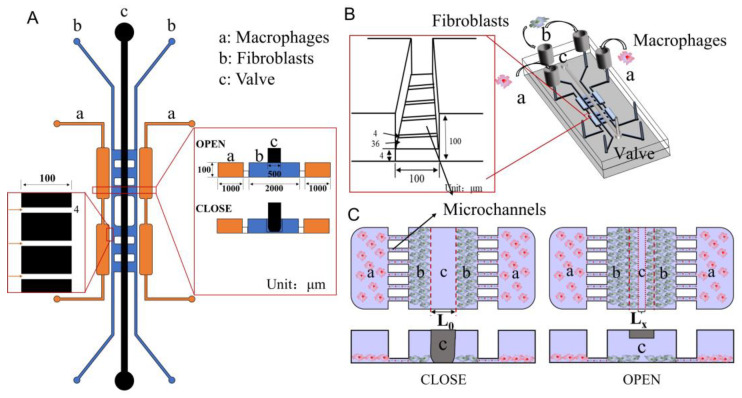
(**A**) 2D design diagram of the microfluidic chip. (**B**) 3D design diagram of the microfluidic chip. (**C**) Schematic diagram of interactions studying between macrophages and fibroblasts.

Herein, a novel microfluidic chip for studying the regulation of skin innate cells by macrophages in different phenotypes has been designed (Scheme 1A,B). The chip is divided into the cell culture layer and the air valve layer. On the lower level (cell culture layer), fibroblasts and macrophages were cultured in the middle and side channels, respectively. Between the channels are extremely tiny microchannels, which are used to prevent the passage of cells and allow the exchange of secretions between cells. The video shows that when the cells flowed into the chip, they didn’t pass through the microchannels (Video S1). When the air valve is closed, the underlying fibroblasts are separated due to pressure to simulate wound formation. After adding macrophages on both sides and opening the air valve, the effect of different macrophages on fibroblasts can be observed in real-time (Scheme 1C). In a word, using this co-culture system, interaction mechanisms between cells can be studied.

## 2. Materials and Methods

### 2.1. Cell Culture

The macrophage cell line RAW264.7 (ATCC TIB-71, USA), and fibroblast cell line NIH-3T3 (ATCC CRL-1658, USA) were used as cell models. The 293T cells (CRL-3216) were used for coating the lentivirus. All cell types were cultured in Dulbecco’s modified eagle medium (DMEM) supplemented with 10% fetal bovine serum (FBS) and 1% penicillin-streptomycin (10,000 U/mL). For differentiation, RAW 264.7 macrophage cells were stimulated with IFN-γ (10 ng/mL) and LPS (40 ng/mL) for 24- hours to obtain M1 macrophages or IL-4 (40 ng/mL) for M2 polarization [28,29,30].

### 2.2. Construction of Fluorescent Cells

RAW264.7 was modified with red fluorescence (mCherry) and NIH-3T3 with green fluorescence (GFP) through genetic engineering technology. When the density of the 293T cells in the 6-well plates was about 60%, the serum-free medium was hanged 1 h before transfection. Plasmid including 2 μg CMV-GFP or CMV-mCherry and 1.5 μg psPAX2, 0.5 μg pMD2.G was added into a 200 μL serum-free medium. Then 8 μL TurboFect was added and mixed well, incubated at RT for 20 min. The mixed system was transferred to the 293T cell medium and incubated at 37 °C, 5% CO2 for 8 h. After post-transfection, the medium was replaced with a 20% serum concentration medium.

At 48 h post-transfection, the supernatant was collected and 0.1% polybrene was added. When the density of the RAW264.7 or NIH-3T3 cells was about 60%, the medium was changed and all the virus solution was added. The medium was changed at 24 h post-infection, and then 0.1% puromycin was added at 48 h. Screen to no cell death, and the GFP-labeled NIH-3T3 cells and mCherry-labeled RAW264.7 cells were constructed.

### 2.3. Fabrication of the Microfluidic Chip

The proposed microfluidic chip was fabricated based on the multi-layer soft lithography as described previously [31]. Briefly, using standard lithographic procedures, a two-level SU-8 master mold was fabricated to create the cell culture layer including two macrophage channels (marked with an in Scheme 1) and two fibroblast channels (marked with b in Scheme 1). In addition, a single-level SU-8 master mold was fabricated to create the air valve layer. After the preparation of molds, a replica molding process was performed to create the poly (dimethyl siloxane) (PDMS) cell culture layer and air valve layer. The upper air valve layer was fabricated by casting a thick layer (3 mm) of PDMS (ratio 15:1 of base material to curing agent, Sylgard^®^ 184, Dow Corning, MI, USA) onto the valve mold, while for the lower cell culture layer, PDMS (10 : 1 ratio) was spin-coated onto the wafer to create a thin layer (~ 200 μm) of microstructured PDMS. Both layers were partially cured for ~20 min at 75 °C and the valve layer was released from the mold and aligned with the cell culture layer. The assembly was again incubated for 1 h at 75 °C to enable off-ratio PDMS bonding. Next, the inlet and outlet ports for both layers were created using a punching tool. Finally, the punched PDMS assembly was sealed with a cover glass after oxygen plasma activation.

### 2.4. Immunofluorescence Staining and Imaging

NIH-3T3 cells were seeded in the middle microchannels of the microfluidic chip (Scheme 1, blue area, marked with b) at the same density, and then M1 or M2 macrophages were seeded to make NIH-3T3 cells co-cultured with different macrophages. A culture medium was added from the side of the macrophages and cultured for 24 h. The cells were washed with PBS once and then fixed with 4% paraformaldehyde at room temperature for 40 min. Subsequently, washed with PBS once, then cells were permeabilized by PBS containing 0.01% TritonX-100 for 20 min. A 10% BSA-PBS solution was used for blocking overnight at 4 °C. On the second day, the primary antibody (anti-α-SMA) was diluted at 1:400 and incubated overnight at 4 °C. After washing 3 times with PBST (PBS + 0.1% Tween-20), the phalloidin (1:400), DAPI (1:400), and the fluorescent secondary antibody at 400:1 dilution were added into the chip and incubated at room temperature and protected from light for 4 h. Immediately afterward, the cells were washed with PBST thrice. Then, the pictures can be taken on the laser scanning confocal microscope. Five independent immunofluorescence assays on five microfluidic chips were carried out simultaneously.

### 2.5. Scratch Assay

The NIH-3T3 cells were plated in six-well plates. When the cells were grown to 80–90% density, a uniform scratch was produced through a sterile pipette tip. The cells were mono-cultured or co-cultured with M1/M2 macrophages which were seeded in the upper transwell chamber (0.4 μm-sized). At 0 h, 12 h, and 24 h after the scratch, the white light of a fluorescent microscope was used to take pictures of the scratch under the 10X objective lens, and 5 positions for each group of samples were taken. The width of the scratch was measured to calculate the relative closure rate (Figure S1). Three independent scratch assays were carried out simultaneously. The area reduction of the scratch assays was calculated using ImageJ software.

### 2.6. Statistical Analysis

All data were expressed as mean ± standard deviations (SD). One-way analysis of variance (ANOVA analysis) and the Student’s *t*-test was used to statistically analyze the data obtained from the experiments. Statistical differences were shown by * (*p* < 0.05), ** (*p* < 0.01), and *** (*p* < 0.001).

## 3. Results and Discussion

### 3.1. Design, Fabrication, and Characterization of the Microfluidic Chip

**Figure 1:**
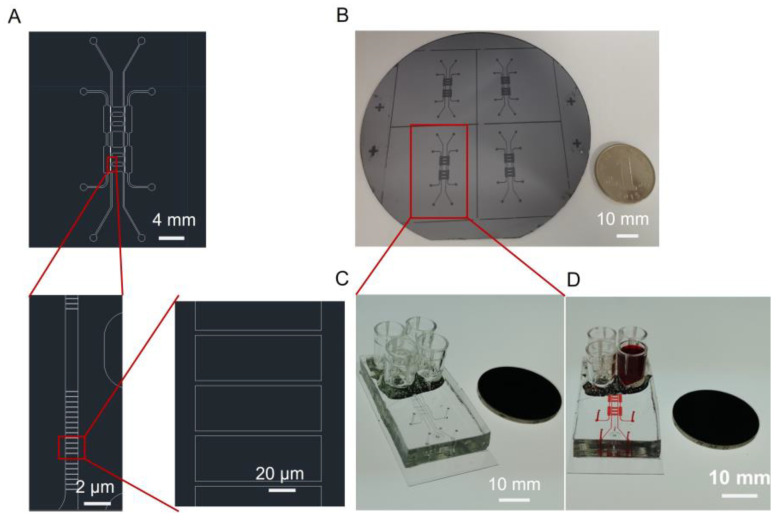
(**A**) Design drawing of the microfluidic chip. (**B**) The positive mode of the microfluidic chip on a silicon wafer. (**C**) Image of the microfluidic chip that consists of PDMS and cover glass. (**D**) The microfluidic chip with microchannels filled with red ink.

Fibroblasts and macrophages are two types of critical cells in the microenvironment of skin wound healing. A microfluidic chip was designed and fabricated to study the interactions between fibroblasts and macrophages that reflect the skin microenvironment in vivo. Scheme 1 presents the design of the chip used in this study. This chip includes two layers: a lower layer for cell culture and an upper layer for air valves. The layer of cell culture contains four units. The two sides of the chip (orange area, marked with a) were used to culture macrophages, while the two middle units (blue area, marked with b) were used to culture fibroblasts and study their biological phenomena. To explore the effect of cytokines secreted by macrophages on fibroblasts, 4.00 μm width channels were designed to connect the orange area and the blue area, which allows the exchange of components in the extracellular culture medium without direct cell contact. The design drawing of the microfluidic chip was presented in Figure 1A. Through two steps of UV lithography and soft lithography, the positive mode was successfully fabricated on a silicon wafer (Figure 1B). PDMS with replicated microchannel from the positive mode was bonded to a cover glass by plasma treatment and the microfluidic chip was obtained (Figure 1C). PDMS is the ideal material for fabricating the chip because it is malleable and basically non-toxic to cells, hence, they are often used to prepare cell culture devices or wearable devices [32,33]. To evaluate the integrity and connectivity of the cell culture channels of the chip, red ink was added to a sample well for observation. As shown in Figure 1D, the red ink evenly filled the entire culture layer channel, which indicated that the chip has good sealing and connectivity.

**Figure 2:**
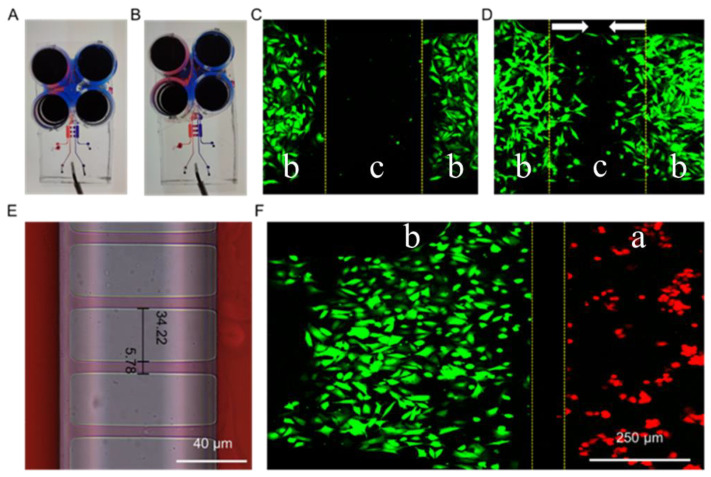
Images of the microfluidic chip with the air valve closed (**A**) and opened (**B**). Fluorescence images of NIH-3T3 cells on the microfluidic chip before (**C**) and after (**D**) valve opened. (**E**) Image of the microchannels connecting part a and part b of the microfluidic chip. (**F**) Fluorescence images of NIH-3T3 (green) and RAW264.7 (red) separated by microchannels connecting part a and part b. “a”, “b” and “c” were marked to indicate different areas of the microfluidic chip. It was consistent with Scheme 1. The arrows in (**D**) indicated the cell migration directions.

There are two critical functional areas on the microfluidic chip: the air valve and the connection channels between macrophages and fibroblasts. When the air was introduced into microchannel c, the pressure was generated and subsequently caused the PDMS under microchannel c to press down. Hence, microchannel b was separated by the pressed PDMS, and the situation was called valve closed (Scheme 1A,C CLOSED). On the contrary, when the air was drawn away from microchannel c, the PDMS under the microchannel c returned to its original shape and the lower channel was connected again. The situation was called valve open (Scheme 1A,C OPEN). When the air valve was closed, the channel in the middle of the cell culture layer was separated (Figure 2A). NIH-3T3 cells were separated to simulate wound formation (Figure 2C). When the air valve was open, the channel was connected (Figure 2B). As shown in Figure 2D, the NIH-3T3 cells grew towards each other which was used to mimic wound healing in vitro.

As another key structure of the microfluidic chip, the precision of the microchannels connecting part a and part b is crucial for studying non-contact or indirect intercellular interactions. Due to errors in the preparation process of the microfluidic chip, the actual size of the microchannels was 5.78 μm (Figure 2E), which is slightly larger than the designed width of 4.00 μm. Furthermore, when the cells were seeded in the chip, the macrophages (RAW264.7, red fluorescence) and the fibroblasts (NIH-3T3, green fluorescence) were well separated (Figure 2F), while the exchange of materials in the extracellular culture medium was not affected (Figure 2E). It should be noted that when the cells were cultured for a long time (three days or more), the cell density was so high that some of the cells passed through the microchannels between both part a and part b, and the same for part b and part b. Therefore, our study was focused on exploring the indirect effect between cells at the early stage of co-culture (12–36 h).

### 3.2. Culture of Macrophages and Fibroblasts in the Microfluidic Chip

**Figure 3:**
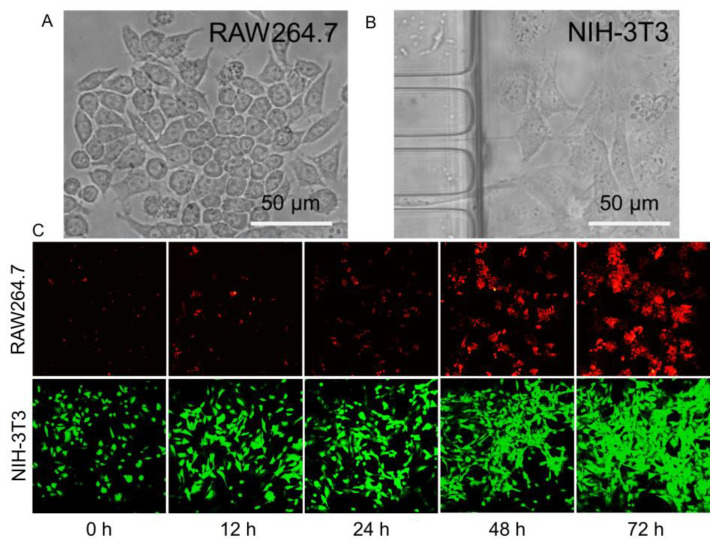
(**A**) and (**B**) Microscope images of macrophages and fibroblasts in white light and the morphology of RAW264.7 and NIH-3T3 cells cultured in the microfluidic chip. (**C**) Fluorescence images of macrophages and fibroblasts with increased incubation time.

Poly-L-lysine (PLL) is widely used to enhance cell adhesion to the solid matrix by enhancing the electrostatic interaction between negatively charged ions on the surface of the cell membrane and the PLL-coated surface. To promote cell adhesion and growth ability in the microfluidic chip [34,35], poly-L-lysine (0.2 mg/mL) was coated on the surface of microchannels. The macrophages and fibroblasts were seeded separately on the microfluidic chips. As shown in Figure 3A,B, the macrophages (RAW264.7) and the fibroblasts (NIH-3T3) were all normal after being cultured in the microfluidic chip for 48 h. In Figure 3C, both fluorescent RAW264.7 and NIH-3T3 were time-dependently increasing. Therefore, the RAW264.7 and NIH-3T3 could proliferate within the space of the corresponding channels over three days, during which their growth and interaction could be monitored and characterized.

The microfluidic chip was reusable after the cell culture. It can be refreshed with trypsin digestion and distilled water cleaning of the cells in the microfluidic chip. Following re-coated microchannels with PLL, it can be reused for the next round of the experiment.

### 3.3. Effect of Macrophages on the Immigration of Fibroblasts

**Figure 4:**
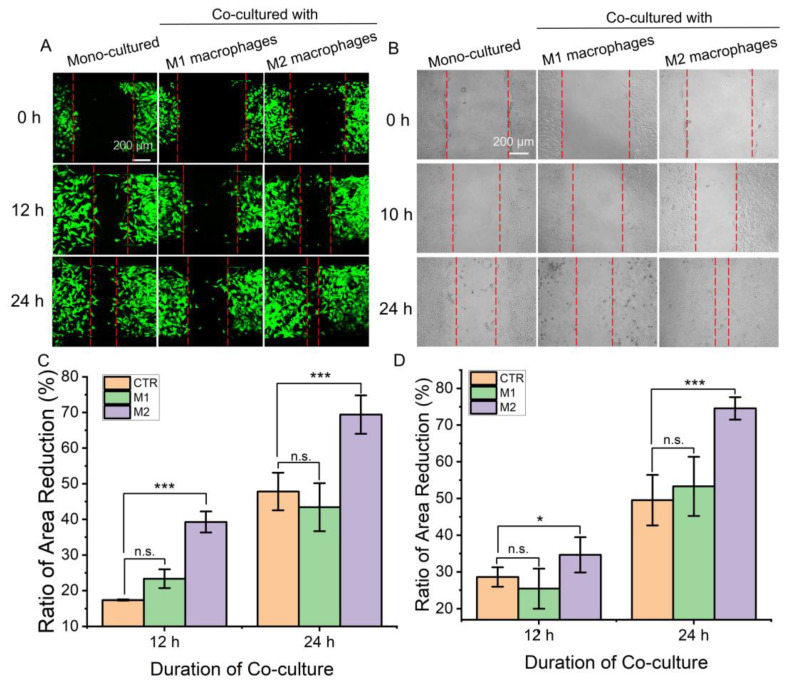
The migration ability of fibroblasts under co-culture with different macrophages. (**A**,**C**) A picture for representative regions showing fibroblast migrated at the septum formed when the air valve closed and a comparison of the area reduction. Measure the reduction of inter-fibroblast septal area relative to 0 h. (**B**,**D**) Images of fibroblast migration at scratches in a conventional transwell co-culture system and comparison of the area reduction. Measure the reduction of scratch area relative to 0 h. Herein, “CTR” refers to mono-cultured, “M1” and “M2” refer to “co-cultured with M1 macrophages” and “co-cultured with M2 macrophages”. Data are presented as mean ± SD (n = 3). “*” is the intragroup comparison with “CTR”. * *p* < 0.05; *** *p* < 0.001.

Fibroblasts play an important role in wound healing by proliferating and migrating to the wound site and being activated to secrete extracellular matrix components to fill wound defects. Macrophages are key cells and function in multiple processes of wound healing. Therefore, it is important to investigate the effect of macrophages on fibroblasts. Different subtypes of macrophages and fibroblasts were inoculated in the corresponding regions of the microfluidic chips. When the air valve was turned off, the fibroblasts were separated by a gap which was used to mimic the wound-healing microenvironment. After the air valve was turned on, the effect of different macrophages on fibroblast migration could be determined by observing the proportion of gap narrowing. As shown in Figure 4A,C, the intercellular septum area of the fibroblasts co-cultured with M2 macrophages decreased the most, indicating that M2 macrophages could promote the migration of fibroblasts compared with M1 macrophages or mono-cultured. Then, conventional co-culture systems and scratch experiments were used to verify the results. Similarly, fibroblasts co-cultured with M2 macrophages showed the highest reduction rate of scratch spacing (Figure 4B,D). Results demonstrated that M2 macrophages can promote the migration of skin fibroblasts and wound healing.

Although results from both methods reveal the same conclusion, the microfluidic chip-based assay showed its advantages. The entire experimental process can be conducted in the microfluidic chip and cell migration can be observed and recorded dynamically in real-time. The experimental demand for cells was low and conducive to the observation of single cells. Meanwhile, the obtained results are conducive to effective statistical analysis compared with the scratch experiments, possibly because the experimental operation is highly controllable in the microfluidic chip [21]. Moreover, the intercellular substance exchange and signal communication between macrophages and fibroblasts was bidirectional, which was closer to the in vivo microenvironment.

### 3.4. Effect of Macrophages on Activation of Fibroblasts

**Figure 5:**
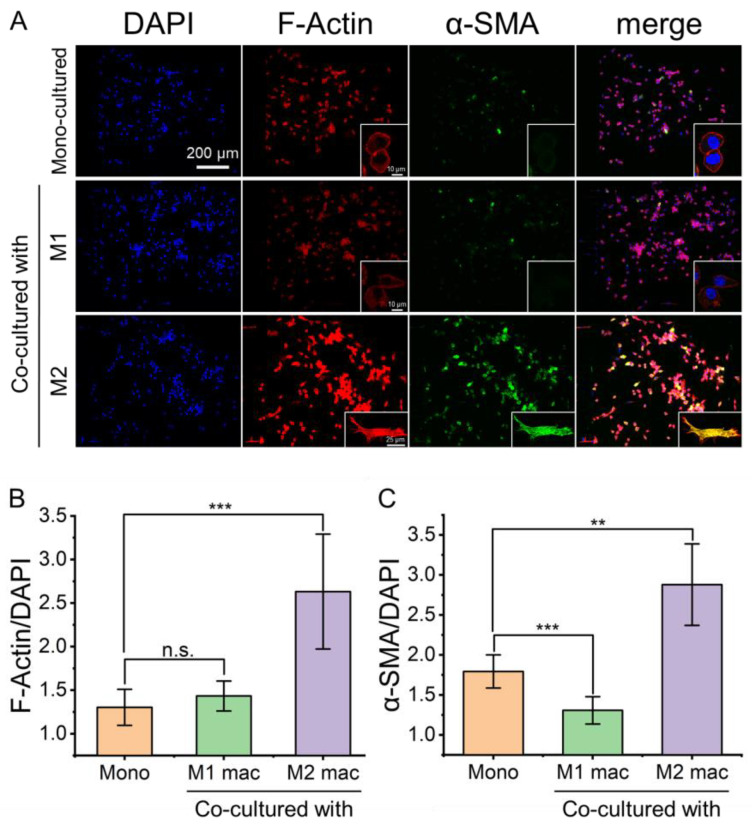
Fluorescence images of fibroblasts with stained α-SMA and F-actin to reveal the activation of fibroblasts by co-culture with different macrophages. (**A**) Fluorescence images of fibroblasts stained for F-actin and α-SMA showing differential expression levels under mono- and co-culture conditions. (**B**,**C**) the ratio of F-Actin/DAPI and α-SMA/DAPI. Herein, “Mono” refers to mono-cultured, “M1 mac” and “M2 mac” refer to “co-cultured with M1 macrophages” and “co-cultured with M2 macrophages”, respectively. Data are presented as mean ± SD (n = 5). “*” is the intragroup comparison with “Mono”. ** *p* < 0.01; *** *p* < 0.001.

Fibroblast migration is closely related to its activation. When the fibroblasts were co-cultured with M2 macrophages, the expression levels of the F-actin and α-smooth muscle actin (α-SMA) were significantly changed. Compared with mono-cultured or co-cultured with M1 macrophages, the fibroblasts co-cultured with M2 macrophages showed signs of activation, and the expression levels of α-SMA and F-actin were increased (Figure 5A). F-actin stress fibers showed the elongated spindle shape of fibroblasts (Figure 5A). The ratio of F-Actin/DAPI (Figure 5B) and α-SMA/DAPI (Figure 5C) of mono-cultured fibroblasts, fibroblasts co-cultured with M1 macrophages, and fibroblasts co-cultured with M2 macrophages were calculated quantitatively. It showed that the expression levels of F-actin and α-SMA were significantly up-regulated when the fibroblasts were co-cultured with M2 macrophages for 24 h. It revealed that M2 macrophages can activate fibroblasts further to promote wound healing.

## 4. Conclusions

In this study, a microfluidic chip was developed to simulate an in vitro co-culture model of macrophages and fibroblasts. The microfluidic chip is without direct macrophages–fibroblasts contact, in which soluble factors can be transported across the medium channel (5.78 μm) and the potential development of local concentration gradients may occur. Subsequently, we demonstrated that macrophages and fibroblasts could be co-cultured using this microfluidic chip to simulate mutual microenvironmental interactions. Air valves were used to isolate fibroblasts to mimic wound formation, and M2-type macrophages were found to promote the migration and activation of fibroblasts, thereby accelerating wound healing. The on-chip results were also verified by a conventional co-culture system. Immunofluorescence staining results showed that M2-type macrophages could promote the expression of A-SMA and F-actin in fibroblasts. In short, the microfluidic chip can mimic the wound healing microenvironment. M2-type macrophages were identified to promote the activation and migration of fibroblasts, thereby accelerating wound healing. In addition, cell migration and cell–cell interactions are crucial in tumor formation, embryonic development, and a variety of other biological processes. The developed microfluidic chip could be easily expanded to these fields of research as a technology or a platform.

## Data Availability

Not applicable.

## Supplementary Materials

The following supporting information can be downloaded at: https://www.mdpi.com/article/10.3390/bios13010070/s1, Figure S1. The scheme of the transwell experiment; Video S1: Support information video 1.
