# Supplementary material for: A Microfluidic Cell Co-Culture Chip for the Monitoring of Interactions between Macrophages and Fibroblasts
Source: Biosensors (Basel). 2022 Dec 31;13(1):70. doi: 10.3390/bios13010070 (PMC9855520; doi:10.3390/bios13010070)
Supplement: Supplementary file 1 [file biosensors-13-00070-s001.zip › biosensors-2028190-supplementary.pdf]

Supporting Information

A Microfluidic Cell Co-Culture Chip for the Monitoring of Interactions between Macrophages and Fibroblasts

Pengcheng Li [1,†], Feiyun Cui [2,†], Heying Chen [3,†], Yao Yang [4,5], Gang Li [6], Hongju Mao [7,*] and Xiaoyan Lyu [4,5,*]

[1] Department of Orthopedics, West China Hospital, West China School of Nursing, Sichuan University, Chengdu 610041, China
[2] School of Basic Medical Sciences, Harbin Medical University, Harbin 150081, China
[3] The Ministry of Education Key Laboratory of Clinical Diagnostics, School of Laboratory Medicine, Chongqing Medical University, Chongqing 400016, China
[4] Department of Dermatology, West China Hospital, Sichuan University, Chengdu 610041, China
[5] Laboratory of Dermatology, Clinical Institute of Inflammation and Immunology, Frontiers Science Center for Disease-related Molecular Network, West China Hospital, Sichuan University, Chengdu 610041, China
[6] Key Laboratory of Optoelectronic Technology and Systems, Ministry of Education, Defense Key Disciplines Lab of Novel Micro-Nano Devices and System Technology, Chongqing University, Chongqing 400044, China
[7] State Key Laboratory of Transducer Technology, Shanghai Institute of Microsystem and Information Technology, Chinese Academy of Sciences, Shanghai 200050, China
* Correspondence: hjmao@mail.sim.ac.cn (H.M.); lxiaoyan@scu.edu.cn (X.L.)
† These authors contributed equally to this work.

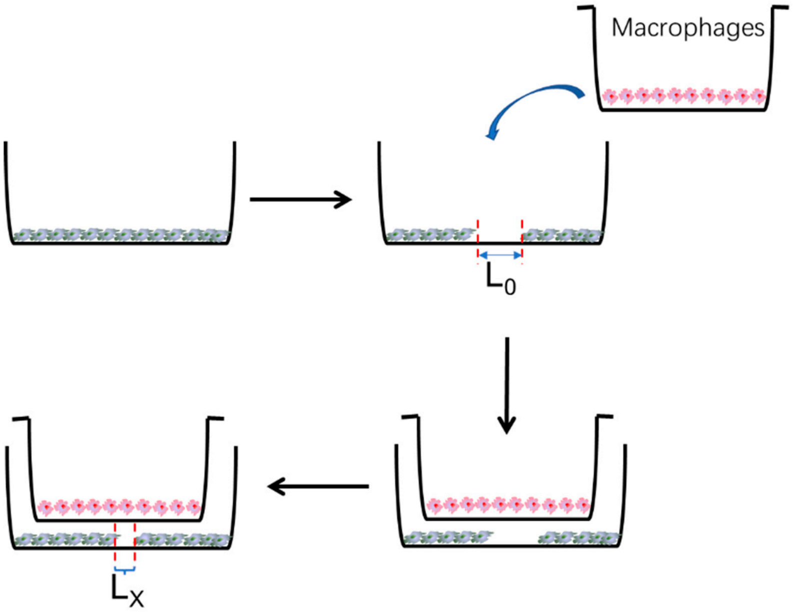

Figure S1. The scheme of the transwell experiment.
